# Oxalic Acid as a Hydrogen Donor for the Hydrodesulfurization of Gas Oil and Deoxygenation of Rapeseed Oil Using Phonolite-Based Catalysts

**DOI:** 10.3390/molecules25163732

**Published:** 2020-08-15

**Authors:** José Miguel Hidalgo Herrador, Jakub Fratczak, Zdeněk Tišler, Hector de Paz Carmona, Romana Velvarská

**Affiliations:** Unipetrol výzkumně vzdělávací centrum, a.s., Research Department, Revoluční 1521/84, 400 01 Ústí nad Labem, Czech Republic; jakub.fratczak@unicre.cz (J.F.); zdenek.tisler@unicre.cz (Z.T.); hector.carmona@unicre.cz (H.d.P.C.); romana.velvarska@unicre.cz (R.V.)

**Keywords:** hydrodesulfurization, deoxygenation, phonolite, oxalic acid

## Abstract

The use of renewable local raw materials to produce fuels is an important step toward optimal environmentally friendly energy consumption. In addition, the use of these sources together with fossil fuels paves the way to an easier transition from fossil to renewable fuels. The use of simple organic acids as hydrogen donors is another alternative way to produce fuel. The present work reports the use of oxalic acid as a hydrogen donor for the catalytic hydrodesulfurization of atmospheric gas oil and the deoxygenation of rapeseed oil at 350 °C. For this process, one commercial NiW/SiO_2_–Al_2_O_3_ solid and two NiW/modified phonolite catalysts were used, namely Ni (5%) W (10%)/phonolite treated with HCl, and Ni (5%) W (10%)/phonolite treated with oxalic acid. The fresh phonolite catalysts were characterized by Hg porosimetry and N_2_ physisorption, ammonia temperature programmed desorption (NH_3_-TPD), X-ray diffraction (XRD), and X-ray fluorescence (XRF). The sulfided metal phonolite catalysts were characterized by XRD and XRF. Hydrodesulfurization led to a decrease in sulfur content from 1 to 0.5 wt% for the phonolite catalysts and to 0.8 wt% when the commercial catalyst was used. Deoxygenation led to the production of 15 and 65 wt% paraffin for phonolite and commercial solids, respectively. The results demonstrate the potential of using oxalic acid as a hydrogen donor in hydrotreating reactions.

## 1. Introduction

The use of renewable available from local sources to produce fuels is an important step toward minimizing the use of fossil fuels, with the use of simple organic acids as hydrogen donors representing a possible option. Oxalic and formic acids produced from raw biomaterials can be considered renewable options to be used for hydrogen-transfer reactions. The use of formic acid for this type of reaction has been extensively studied [1,2,3] in which oxygen free aromatics are produced from lignin or other hydroconversion reactions. By comparison, oxalic acid has been less frequently used by researchers but has great potential to be used as a renewable hydrogen donor because it can be produced from carbohydrate waste and microorganisms, with one example being the oxalic acid produced from corncobs using *Aspergillus niger* (via semi solid-sate fermentation) [4,5]. Although the price of hydrogen gas per kg is lower than that of hydrogen obtained by oxalic acid decomposition [6], the use of oxalic acid can be justified when considering that oxalic acid is solid, rather than a gas or liquid (as in the case of formic acid), and allows for the production of hydrogen from a solid source, which may either be required or a useful option in some cases. Formic acid is used as a hydrogen donor but is a more complicated compound to manage due to its toxicity, irritability, and bad odor. By contrast, oxalic acid is a relatively clean source of hydrogen, yielding CO_2_, CO, and H_2_O. Interest in the use of oxalic acid is also related to its suitability for reactions performed in closed (hermetically) reactors without the need to add gases prior to reaction.

Furthermore, the revised Renewable Energy Directive (EU) 2018/2001 aims to increase the use of energy from renewable sources in the European Union. At present, fossil fuels continue to be used worldwide on a massive scale. Thus, the co-processing of renewable compounds with fossil feedstocks (crude oil, for example) represents a practical option for an easier transition from the use of fossil to renewable fuels as a progressive way of improving the renewability of the overall fuel production process [7,8]. Thus, the use of oxalic acid as a hydrogen source in some refinery processes is an alternative with great potential alternative that needs to be studied.

The co-processing approach applies not only to the hydrodesulfurization (HDS) and hydrodenitrogenation (HDN) of the middle distillates but also to the deoxygenation (DO) of the triglycerides. The deoxygenation and hydrodesulfurization (HDS) processes have been previously described in the literature [9,10]. The reactions need to be carried out under hydrogen pressure to remove oxygen and sulfur compounds from the triglycerides and atmospheric gas oil (e.g., by producing water, CO—decarbonylation, CO_2_—decarboxylation of the triglycerides (deoxygenation products), or H_2_S (HDS)).

Commercial hydrotreating catalysts that use Al_2_O_3_ and SiO_2_–Al_2_O_3_ as supports are commonly used in many refinery processes, such as the hydrodeoxygenation of triglycerides [11,12]. Phonolite (Ph) ore is a cheap mineral rich in Al_2_O_3_ and SiO_2_ that can be used as a raw material for catalyst production, especially given that phonolite ore was previously tested for use in hydrotreating reactions [12] and is rich in SiO_2_–Al_2_O_3_ like the other standard catalysts used to produce renewable fuels [13]. Phonolite is also a locally available resource in Northwest Czech Republic that is already being extracted for other purposes, e.g., as a fluxing agent in ceramic masses and glass batches, where coloring oxide content is not an issue [14].

In this work, the hydrotreatment of atmospheric gas oil and rapeseed oil (RSO) was tested using oxalic acid as a hydrogen donor with three different NiW/supported catalysts. Here, we describe the main properties of the used catalysts (commercial NiW/SiO_2_–Al_2_O_3_ and two synthesized NiW/acid-modified phonolite catalysts) and evaluate their efficiency during hydrotreatment.

## 2. Results and Discussion

### 2.1. Solids Characterization

X-ray fluorescence (XRF) was used to determine the elemental composition of all solids. The results are presented in Table 1. Phonolite treatment with acids (HCl and oxalic acid) indicated a reduction in the Al, Na, Fe, and Ca contents. The results for Ni and W contents (impregnated materials) were as expected, with the NiW/Ph-HCl solids containing higher contents of Ni and W compared to the NiW/Ph-Ox catalyst. The acid treatments resulted in solids with a higher porosity, with the Ph-HCl support being the most porous material.

**Figure 1:**
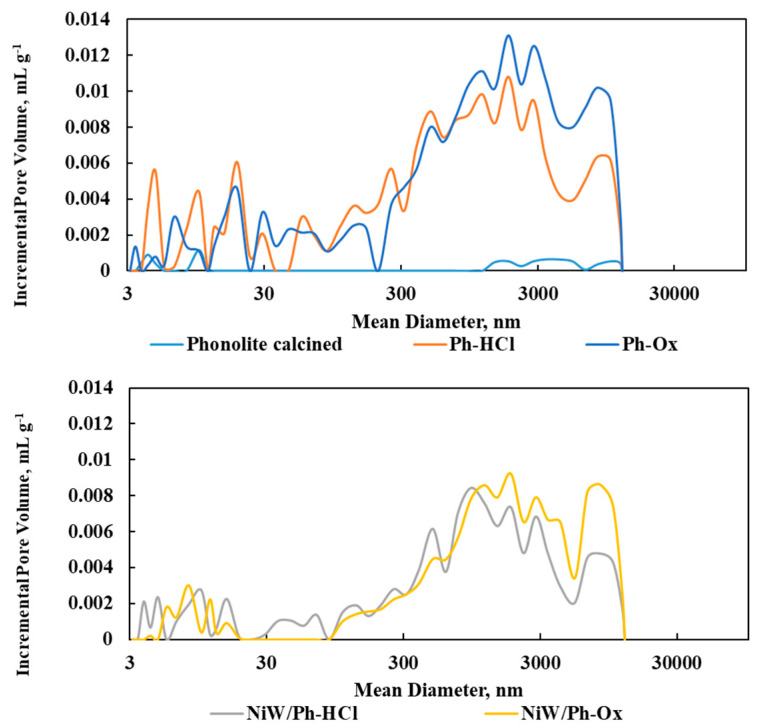
Hg porosimetry of the original phonolite material and its derived solids.

The basic raw material for the preparation of the catalysts (phonolite) has very poor textural properties, and its treatment with acid led to rapid changes in the material’s textural properties (an increase in pore volume and specific surface area). Acid leaching with oxalic acid resulted in a higher total pore volume than the sample leached with HCl. On the other hand, a higher volume of “mesopores” (i.e., pores with dimensions of 3 to 50 nm) can be seen here (Table 2, Figure 1). The measured parameters for catalysts prepared on the basis of these modified phonolite supports were all lower in value, which is related to the blocking of the pores by the deposited active metals (Ni and W).

**Figure 2:**
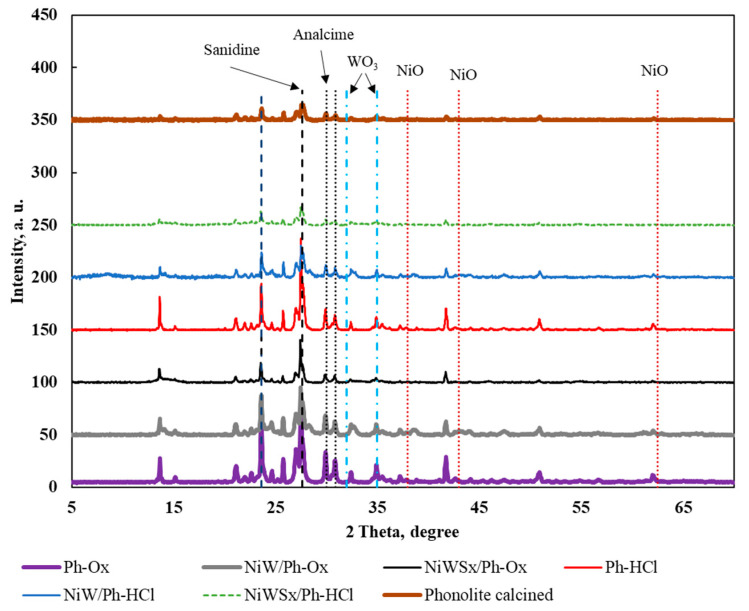
Wide angle X-ray diffraction (XRD) patterns of the phonolite solids.

X-ray diffraction (XRD) patterns were acquired to obtain more information about the materials’ structures. The XRD diagrams (Figure 2) were similar for all solids (i.e., the signals had the same angle). However, only peaks related (XRD patterns) to minerals from the feldspar group were clearly found (sanidine ((PDF 19-1227), analcime (PDF 72-0445), and nepheline (PDF 70-1582) diagrams) [15].

There was no evidence of NiO crystal phase for the solids containing Ni at 2*θ* about 38° (111), 43° (200), and 62.5° (220) [16]. In addition, the diffraction peaks at 2*θ* = 32 and 35° assigned to the reflections associated with tungsten oxide (WO_3_, (220)) [17] were not clear. This shows that the active phase is very well distributed on the support surface.

**Figure 3:**
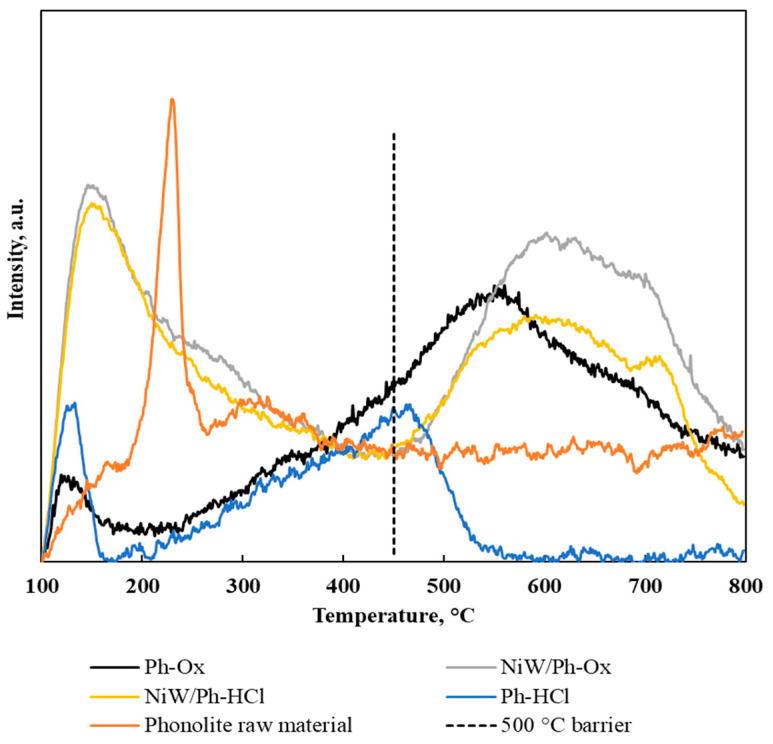
Ammonia temperature programmed desorption (NH_3_-TPD) profiles of phonolite modified supports and the maxima of the TPD signals with each particular percentage contribution.

Ammonia temperature programmed desorption (NH_3_-TPD) characterization was carried out to study the acidity of the supports prior to being impregnated (Figure 3). Ph-HCl presented two main zones of desorption peaks: the first at 100–400 °C and the second at 400–800 °C. The peaks from 100 to 250 °C correspond to weak and intermediate acid sites, those from 250 to 400 °C correspond to intermediate acid sites, and those at >400 °C correspond to strong acid sites. PhOx presented more strong acid sites than Ph-HCl, with the largest number of weak acid sites at 130 °C (100–200 °C temperature zone) and a larger amount of stronger acid sites as represented in the curve from 200 to 800 °C. However, from 450 °C, the observed signals are not only due to the desorption of ammonia, but also to water produced from the dehydroxylation of –OH surface groups.

Ph-Ox presented a larger number of strong acid sites compared to Ph-HCl. However, Ph-HCl presented many more weak–intermediate acid sites than Ph-Ox. Thus, these two supports presented different acidity values, as shown in Figure 3. The total amount of desorbed ammonia for the temperature up to a maximum of 450 °C is shown in Table 3.

### 2.2. Hydrotreating Tests Results

The DO and HDS mass balance results are shown in Table 4. Large-scale production of gases was found for all the tests due to the decomposition of oxalic acid to gases and water (CO + CO_2_ + H_2_O). This decomposition has previously been reported [5,18,19,20,21,22]. In our case, oxalic acid decomposition appeared to yield only CO_2_ and H_2_, as shown in Table 5. However, the CO that was being produced after each decomposition was rather reacting with water, producing CO_2_ and H_2_ [23]. Even some oxygenated compounds were created and retained in the aqueous phases (liquid products) and in the solid products (Table 2).

For DO products, an organic phase immiscible with a small amount of the aqueous phase was produced. Some solids were also produced when the phonolite-derived catalysts were used. No solids were found for the commercial catalysts compared to the solids produced by the two phonolite-derived catalysts. This solid production could be due to the reaction of glycerol [24]. Here, glycerol can be transformed into propane and propylene, and into carbonaceous solids when phonolite catalysts are present, in which case the gaseous products include lower amounts of C3 gases (propylene, propane) (Table 5). The higher Ni and W contents in the SiO_2_–Al_2_O_3_ commercial material could explain the lower production of solid products. The production of aqueous phase was due to water production.

For the HDS tests, the amount of aqueous phase was higher compared to the DO tests, which presented a lower wt% of organic liquid products. Solid products were found for all HDS tests—in this case, presenting a higher quantity of solid products for the commercial catalyst but a lower amount of the product compared to the aqueous phase, indicating the possible production of polar compounds (water and others) that favor the final production of solids. Another reason for solid carbonaceous production is the commercial catalyst’s greater cracking capacity, resulting from its higher tungsten content, which thereby provides higher content of gases in its products, with more methane and less hydrogen. This is indicative of a hydrocracking reaction, with the gas production indicating higher solid production.

**Figure 4:**
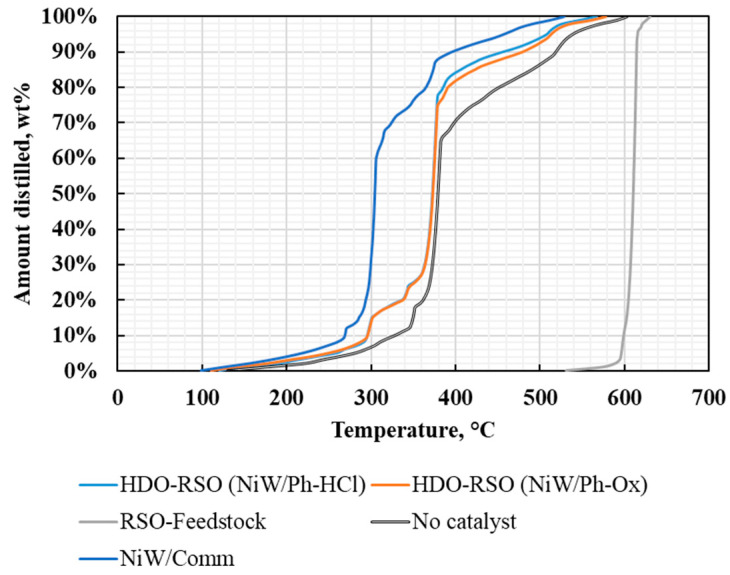
Simulated distillation (SimDis) for the deoxygenation (DO) products and feedstock.

The simulated distillation (SimDis) (Figure 4, Table 5) of the organic liquids for the DO tests indicated that phonolite-derived materials were able to catalyze the production of only 17–18 wt% of paraffin (C17) and 4 wt% of lighter compounds from the RSO oil. The commercial catalyst was able to catalyze the production of 69 wt% of C17 compounds, with 5 wt% of other lighter compounds. Thus, the commercial solid was more capable of deoxygenating the RSO oil than the phonolite materials, which presented similar activity (NiW/Ph-HCl and NiW/Ph-Ox).

For the HDS tests, an elemental analysis for % C, H, N, and S of the organic liquid phase was performed, in which similar results were obtained for the hydrogen and carbon contents (Table 6). This is the first time that oxalic acid was used for the HDS reaction. In this case, the atmospheric gas oil was used as feedstock, and the HDS decreased from 1 wt% to 0.2 and 0.5 wt% for tests using commercial and phonolite catalysts, respectively. The sulfur contents differed for each product depending on each test. The highest decrease in sulfur content was found for products using the catalyst NiW/Comm. Phonolite catalysts presented a similar sulfur decrease (0.56 and 0.52 wt% sulfur content for NiW/Ph-HCl and NiW/Ph-Ox, respectively, compared to 1 wt% sulfur content in the feedstock).

The density decreased in all cases after the HDS tests, with the product obtained from the NiW/Comm catalyst having a lower density. This product also presented the lowest refractive index. As shown in the literature, the refractive index and density are closely related properties [19].

**Figure 5:**
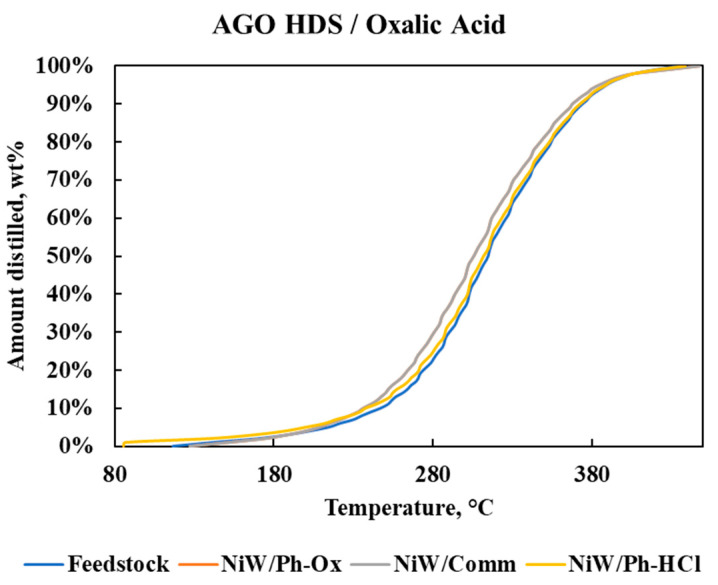
SimDis for the hydrodesulfurization (HDS) products and feedstock.

The SimDis results (Figure 5) were similar for the feedstock and products, with the products having a slightly higher content of lighter compounds. The phonolite catalysts presented similar SimDis results, and NiW/Comm presented the highest increase in light compounds due to cracking during the reaction, producing not only methane (Table 5), but also other lighter compounds, as shown in Figure 5 (and having the greatest amount of solid products).

The gaseous products (Table 7) were mainly CO_2_ and hydrogen. Methane, ethane, propane, and propylene were found as secondary products. An unusually high content of propane and propylene was found under the DO tests using NiW/Comm, which could be due to the conversion of glycerol into propane and propylene.

For HDS tests (Table 5), the main gases were CO_2_ and hydrogen, with methane and ethane being the main byproducts. The hydrogen sulfide content was similar for the phonolite catalysts, but low for the test carried out using NiW/Comm, indicating that some sulfur could possibly be transferred to the solid residual product.

## 3. Materials and Methods

### 3.1. Materials and Their Analyses

RSO oil (Table 8) was used in the form of vegetable oil and atmospheric gas oil (AGO) containing 1 wt% sulfur and 0.00179 wt% nitrogen. The AGO had a density of 856.44 kg m^−3^ at 15 °C and a refractive index of 1.4757. Oxalic acid dihydrate (99.5%) (Penta s.r.o. company, Chrudim, Czech Republic) was also used.

The commercial sulfided NiW/SiO_2_–Al_2_O_3_ (NiW/Comm) catalyst employed had 6.4 and 17.5 wt% of Ni and W contents, respectively, and a BET surface area of 200 m^2^ g^−1^, and its catalytic activity was compared with the other two synthesized catalysts. These two catalysts were synthesized using Ph ore as a raw material. This ore was taken from Northwest Czech Republic. The raw Ph material was treated with hydrochloric acid (NiW/Ph-HCl) and oxalic acid (NiW/Ph-Ox) using the same procedure described in the literature [15]. The Ph was supplied in sand form with a size of 0–2 mm by the company Keramost a. s. (Most, Czech Republic). Before catalyst preparation, the phonolite sand was sieved using a Retsch AS300. Twenty grams of phonolite (224–560 μm fraction) was dried at 120 °C overnight. Then, the phonolite was leached using either a 3 M HCl or 1 M oxalic acid solution (80 °C for 4 h). The Ph/acid (g/mL) ratio was 1:10. The product was then filtered, washed (with hot demineralized water), and dried overnight at 120 °C. These dried samples were calcined at 500 °C for 6 h (1 °C min^−1^ at room temperature) in air.

The resulting solids were impregnated with Ni nitrate and ammonium metatungstate to the target Ni and tungsten contents of 5 and 10 wt%, respectively. Finally, the catalyst precursors were calcined for 6 h at 450 °C (1 °C min^−1^) in air. Two laboratory catalysts were created: NiW/Ph-Ox (Ni and tungsten supported on phonolite treated with oxalic acid) and NiW/Ph-HCl (Ni and tungsten supported on phonolite treated with hydrochloric acid).

The composition of the catalysts was ascertained by XRF using an S8 Tiger (Bruker AXS, Advanced X-ray Solutions GmbH, Karlsruhe, Germany) with an Rh cathode. The crystallographic structures of the catalysts were determined by examining the XRD patterns of the powder samples obtained via a D8 Advance ECO (Bruker) applying Cu Kα radiation (λ = 1.5406 Å). A step size of 0.02° and a step time of 0.5 s were used.

The acidic properties of material supports (Ph treated with oxalic and hydrochloric acid) were characterized by NH_3_-TPD temperature programmed desorption using an Autochem 2950 HP (Micromeritics Instrument Corporation, Norcross, GA, USA). In a quartz U-tube reactor, a total of 100 mg of the sample was pretreated in He to 500 °C, with a temperature ramp rate of 10 °C min^−1^. The sample was then cooled to 50 °C and saturated with ammonia at a flow rate of 25 mL/min of 10 vol% NH_3_/He for 30 min. The gas was subsequently changed to helium (25 mL/min) and flushed out until the baseline was continuous (60 min). After this procedure, the temperature increased to 800 °C at a rate of 15 °C min^−1^ to obtain the NH_3_-TPD curves from 100 to 800 °C, with a ramp rate of 15 °C min^−1^. The changes in gas concentration were monitored by a TCD detector. The catalysts tested in the reaction were washed with a cyclohexane/isopropanol mixture at a 70:30 (*v/v*) and then dried at 110 °C for 24 h. The catalysts were then analyzed by XRF.

The BET specific surface area of the solids was determined by N_2_ adsorption/desorption at 196 °C using an Autosorb iQ. Mercury porosimetry measurements were performed on a Micromeritics AutoPore IV 9510 mercury porosimeter (Micromeritics Instrument Corporation, Norcross, GA, USA). All samples were dried prior to their analysis in a glass cell at 110 °C under vacuum for 16 h.

The attenuated total reflectance (ATR) technique was used for the liquid products (DO tests) with a Nicolet iS10-Thermo Scientific instrument (Thermo Fisher Scientific Brno s.r.o., Brno, Czech Republic) (crystal diamond; number of scans = 64; resolution 4 cm^−1^).

### 3.2. Catalytic Tests

An autoclave 4575/76 with a “4848B” controller (Parr Instruments Company, Moline, IL, USA) was used for all tests. The NiW oxide supported catalysts were activated–sulfided using di-t-butyl polysulfide (Lubrizol Company, Wickliffe, OH, USA). In total, 4.2 g of the catalyst and 20 g of di-t-butyl polysulfide were introduced into the reactor. The reactor was pressurized to 50 bar and heated from room temperature to 340 °C (8.3 °C min^−1^) while maintaining the temperature for 2 h. The reactor was then cooled to room temperature and depressurized.

For the DO tests, the autoclave was opened, and 20 g RSO oil, 30 g oxalic acid dihydrate, and 2 g of previously sulfided catalysts were introduced into the reactor. The reactor was then flushed with nitrogen and finally closed at 0 bar (N_2_) to avoid the presence of air. The autoclave was then heated to 350 °C (8.3 °C min^−1^) and this temperature was maintained for 1 h. The autoclave was then cooled via air flow with an average cooling temperature rate of 4.5 °C min^−1^. Finally, a gaseous sample was collected, and the autoclave was opened and weighed to obtain the mass balance (subtracting the final and initial autoclave filled weights). The liquid was then collected, weighed, and filtered (cold filtration).

For HDS tests, the procedure was similar to that carried out for the DO tests (same procedure and temperature) but using 20 g AGO, 30 g oxalic acid dihydrate, and 2 g of the catalyst.

### 3.3. Product Analyses

The mass balance was calculated by the weight of the total filtrated sediment and liquids (cold filtration). Liquid products were centrifuged in 40 mL vials at 2600 rpm for 30 min, thereby obtaining a clean liquid on the upper side with solids + non miscible liquid mixtures at the bottom. Two grams of the initial catalyst amount were subtracted from the total amount of solids to calculate the solid product amounts.

The gas composition was characterized by Agilent’s Refinery Gas Analyzer (RGA) method (Agilent, Santa Clara, CA, USA). Liquid product analyses were as follows: density at 15 °C, measured using a semi-hydrometer KYOTO DA-645 (Kyoto Electronics Manufacturing co., Horiba, Japan), simulated distillation (SimDis) performed by gas chromatography following the ASTM D2887 protocol. Elemental analysis (C/H) was conducted using a FLASH 2000 elemental analyzer (Thermo Fisher Scientific Brno s.r.o., Brno, Czech Republic) (ASTM D5291 protocol), with nitrogen and sulfur content measured at microscale (ppm) using a Trace SN Cube Instrument (Elementar, Langenselbold, Germany) (ASTM D5453 and ASTM D4629 protocols). The refractive indexes of the liquids were determined on an automatic refractometer RFM 970 from Bellingham + Stanley Ltd. (Royal Turnbridge Wells, UK) based on ASTM D1218.

## 4. Conclusions

Three NiW catalysts (one commercial and two synthesized phonolite supports, NiW/Ph-HCl and NiW/Ph-Ox) were used for the hydrotreatment of atmospheric gasoil (AGO) and RSO using oxalic acid as a hydrogen donor. The acid treatment applied to the phonolite support produced a significant change in the material properties, which was especially noticeable in their textural properties. To the best of our knowledge, this is the first study of this kind using real feedstock. In the case of gasoil, significant HDS efficiency was detected. The same behavior was observed in triglyceride deoxygenation, during which partial paraffin formation was detected by SimDis analysis. Our results suggest that it is possible to use oxalic acid as a hydrogen donor instead of external gas flows, which might provide a more sustainable hydrotreatment process without external sources of H_2_.

## Figures and Tables

**Table 1:** X-ray fluorescence (XRF) elemental analysis for the supports, original phonolite material, and catalysts. Hg porosimetry of Ph, Ph-Ox, Ph-HCl, NiW/Ph-Ox, and NiW/Ph-HCl. The elemental composition of NiWSx/Ph-Ox and NiWSx/Ph-HCl sulfided catalysts is also included.

	Ph	Ph-Ox	Ph-HCl	NiW/Ph-Ox	NiW/Ph-HCl	NiWSx/Ph-Ox	NiWSx/Ph-HCl
Si, wt%	26.5	34.0	34.8	27.5	28.5	21.2	12.6
Al, wt%	11.8	7.3	6.7	6.1	5.3	3.0	2.8
Ni, wt%	0.0	0.0	0.0	4.4	5.2	2.2	2.5
W, wt%	0.0	0.0	0.0	9.5	9.8	4.7	5.0
Na, wt%	7.9	2.6	2.8	2.2	1.7	1.0	1.1
K, wt%	5.1	6.9	6.6	6.3	5.2	3.2	3.2
Fe, wt%	1.4	0.7	0.8	0.7	0.5	0.7	0.5
Cl, wt%	0.3	0.0	0.0	0.0	0.02	0.0	0.0
S, wt%	0.0	0.0	0.0	0.0	0.0	22.0	20.8
Others, wt%	0.9	0.1	<0.1	0.5	0.5	0.4	0.3
O, wt%	46.1	48.3	48.3	42.8	43.3	41.6	51.2

**Table 2:** Textural properties of the original phonolite material and its derived solids.

	Ph	Ph-Ox	Ph-HCl	NiW/Ph-Ox	NiW/Ph-HCl
Total intrusion volume (cm^3^ g^−1^) *	0.008	0.201	0.179	0.130	0.118
Pore volume 3–50 nm (cm^3^ g^−1^) *	0.003	0.024	0.030	0.010	0.016
BET surface area, m^2^ g^−1^ **	7.6	114.3	120.1	49.3	53.9

* Hg porosimetry, ** N_2_ physisorption.

**Table 3:** Ammonia temperature programmed desorption (NH_3_-TPD) peak signals and temperatures at each maximum. The total ammonia desorbed at each deconvoluted peak is indicated as “Total”.

**Phonolite Calcined**	Peak Number	1	2	3	Total
	Temperature at Maximum, °C	163	225	235	
	Quantity, NH_3_ mmol g^−1^	0.001	0.031	0.008	0.039
**Ph-HCl**	Peak Number	1	2	3	Total
	Temperature at Maximum, °C	123	134	194	
	Quantity, NH_3_ mmol g^−1^	0.021	0.005	0.000	0.027
**Ph-Ox**	Peak Number	1			Total
	Temperature at Maximum, °C	119			
	Quantity, NH_3_ mmol g^−1^	0.011			0.011
**NiW/Ph-Ox**	Peak Number	1	2		Total
	Temperature at Maximum, °C	149	270		
	Quantity, NH_3_ mmol g^−1^	0.087	0.016		0.103
**NiW/Ph-HCl**	Peak Number	1	2		Total
	Temperature at Maximum, °C	146	268		
	Quantity, NH_3_ mmol g^−1^	0.079	0.030		0.109

**Table 4:** Deoxygenation (DO) and hydrodesulfurization (HDS) mass balance of the products.

**DO**	**NiW/Comm**	**NiW/Ph-HCl**	**NiW/Ph-Ox**
Gas, wt%	46.6	44.2	54.6
Organic liquid, wt%	51.6	44.7	29.8
Aqueous liquid + losses, wt%	1.8	2.1	7.6
Solid, wt%	0	9.0	8.0
**HDS**	**NiW/Comm**	**NiW/Ph-HCl**	**NiW/Ph-Ox**
Gas, wt%	52.2	45.9	44.4
Organic liquid, wt%	23.8	31.1	31.2
Aqueous liquid + losses, wt%	9.4	17.6	17
Solid, wt%	14.6	5.4	7.4

**Table 5:** Simulated distillation (SimDis) results of the DO tests and feedstock.

Boiling Range	<220 °C	220–340 °C	340–420 °C	>420 °C
Feedstock wt%	0	0	0	100
NiW/Comm wt%	5	69	19	7
NiW/Ph-Cl wt%	4	18	65	13
NiW/Ph-Ox wt%	4	17	64	15
No catalyst wt%	2	10	63	25

**Table 6:** C, H, N, and S elemental analysis, density, and refractive index for the organic liquid products of the HDS tests.

C, H, N, S Elemental Analysis	NiW/Comm	NiW/Ph-HCl	NiW/Ph-Ox	Feedstock
N, g kg^−1^	274	177	167	171
S, wt%	0.22	0.56	0.52	1.00
C, wt%	86.6	86.5	86.8	86.8
H, wt%	13.4	13.5	13.1	13.1
Density at 15 °C (kg m^−3^)	849.81	851.74	851.48	856.44
Refractive index at 20 °C	1.47291	1.47346	1.47344	1.47567

**Table 7:** Refinery Gas Analysis methodology (RGA) analyses for gases obtained from the DO and HDS tests using the commercial and laboratory catalysts.

**DO Tests (Gaseous Products)**			
	**NiW/Comm**	**NiW/Ph-HCl**	**NiW/Ph-Ox**
**Compound**	**Mol%**	**Mol%**	**Mol%**
Hydrogen	11.33	15.84	16.21
CO_2_	84.96	81.67	81.24
Methane	0.53	0.39	0.43
Ethane	0.7	0.33	0.37
Propane	1.11	0.1	0.1
Propylene	1.02	0.34	0.35
Others	0.35	1.33	1.3
**HDS Tests (Gaseous Products)**			
	**NiW/Comm**	**NiW/Ph-HCl**	**NiW/Ph-Ox**
**Compound**	**Mol%**	**Mol%**	**Mol%**
Hydrogen	21.15	23.96	23.67
CO_2_	77.63	75.47	75.82
Methane	0.53	0.14	0.17
Ethane	0.25	0.03	0.04
Propane	0.01	0	0
H_2_S	0.03	0.18	0.19
Propylene	0.05	0.03	0.06
Others	0.35	0.19	0.05

**Table 8:** Boiling range distribution of the atmospheric gas oil.

Boiling Range	Amount Distilled, wt%
<180 °C	3
180–280 °C	20
280–350 °C	55
>350 °C	22
